# Enhanced Ambient Stability of Efficient Perovskite Solar Cells by Employing a Modified Fullerene Cathode Interlayer

**DOI:** 10.1002/advs.201600027

**Published:** 2016-03-22

**Authors:** Zonglong Zhu, Chu‐Chen Chueh, Francis Lin, Alex K.‐Y. Jen

**Affiliations:** ^1^ Department of Materials Science and EngineeringUniversity of WashingtonSeattleWA98195‐2120USA; ^2^ Department of ChemistryUniversity of WashingtonSeattleWA98195‐2120USA

**Keywords:** air stability, fullerene, perovskite, solar cells, surfactant

## Abstract

**A novel fullerene cathode interlayer** is employed to facilitate the fabrication of stable and efficient perovskite solar cells. This modified fullerene surfactant significantly increases air stability of the derived devices due to its hydrophobic characteristics to enable 80% of the initial PCE to be retained after being exposed in ambient condition with 20% relative humidity for 14 days.

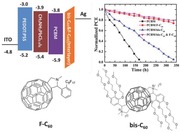

The rapid development of organic–inorganic hybrid perovskite solar cells (PVSCs) has recently attracted the worldwide attention because their power conversion efficiency (PCE) has risen from 4% to >20% within just five years.^1^ This coupled with low cost starting materials and good solution processability has enabled them to be considered as a favorable candidate for next generation photovoltaic technology.^2^ The appealing characteristics of hybrid perovskites also include intense absorption, appropriate direct band gaps, high charge carrier mobility, long charge carrier diffusion length, in addition to facile solution processability.^3^

Currently, the record high PCE (20.1%) of PVSC was demonstrated in a conventional n‐i‐p device configuration, where a mesoporous n‐type transition metal oxide (TiO_2_) capped with perovskite absorber was used in conjunction with an organic hole‐transporting layer (HTL).^4^ However, the fabrication of this superstructured architecture is quite elaborated; therefore, thin‐film PVSCs have also been vigorously pursued and showed comparable high PCEs.^5^ Among the thin‐film PVSCs developed so far, the inverted p‐i‐n device structure with the configuration of indium tin oxide (ITO)/poly(3,4‐ethylenedioxythiophene) polystyrene sulfonate (PEDOT:PSS)/perovskite/phenyl‐C_61_‐butyric acid methyl ester (PCBM)/electrode has attracted the most attention due to its completely low‐temperature processing and less hysteretic *J–V* behaviors, where the PEDOT:PSS serves a HTL and PCBM serves an electron‐transporting layer (ETL).^6^

It is worth to note that due to the polycrystalline nature of the solution processed perovskite film, its resulting morphology, crystallization, and conditions of crystal boundary are strongly correlated with the employed interlayers in device.^7^ Besides, for PVSCs with the stratified configuration, it is important to control the appropriate electrical properties at each interface in the device in order to facilitate charge collection while minimizing the charge recombination.^8^ This necessitates the understanding of the electronic structure and energy level alignment at the metal electrode/charge‐transporting layer (CTL)/perovskite interfaces. To achieve this goal, interface engineering has become very important for improving the photovoltaic performance and stability of PVSCs.^9^

The nature of the electrical contact at electrode/CTL/perovskite interfaces is very complicated since a wide variety of interfacial effects including charge transfer, Fermi‐level tuning, dipole formation, generation of defect states, etc. can happen depending on the type and strength of interactions between materials and the order of contact formation between organic and inorganic interfaces.^10^ Thus far, various interfacial engineering approaches have been applied to PVSCs to promote charge transport and electrode selectivity, such as passivating perovskite/CTL interfaces, introducing cathode buffer interlayer, and doping CTLs.^11^ Another major challenge in interface engineering is to enhance the ambient stability of PVSCs for long‐term uses. This can be solved potentially by using a robust interlayer to increase the stability of the derived device against oxygen and moisture.^12^ For example, it has been documented that the degradation of thin‐film PVSCs with fullerene derivative based electrode geometry is faster than that of the MAPbI_3_ perovskite layer itself.^13^ This might stem from the incomplete coverage of PCBM on perovskite film which results in increased moisture absorption.^13^ Therefore, it is important to improve the ambient stability of fabricated PVSCs by precise controlling of the perovskite/fullerene/electrode interface.^14^

In this work, a novel fullerene derivative (*N*‐methyl‐2‐(2‐perflurooctylphenyl)‐3,4‐fullero‐pyrrolidine, F‐C_60_) with a perfluoroalkyl side‐chain (as shown in **Figure** **1**A) was developed and combined with our previously reported bis‐C_60_ surfactant (*N*‐methyl‐2‐(2,3,4‐tris(2‐(2‐methoxyethoxy)ethoxy) phenyl)‐3,4‐fulleropyrrolidium iodide, bis‐adduct)^15^ to form a robust and efficient cathode interlayer for addressing the stability issues encountered in thin‐film PVSCs. It simultaneously enhances the performance and ambient stability of the fabricated thin‐film PVSCs. When this new hybrid fullerene cathode interlayer (FCI) was used, the derived PVSC showed an increased PCE from 12.1% (without FCI) to 15.5% under the AM 1.5G illumination. More encouragingly, benefitting from the hydrophobicity introduced by F‐C_60_, the device showed a much better ambient stability, retaining 80% of its initial PCE even after being exposed in ambient condition with 20% relative humidity for 14 days. On the contrary, the control device without FCI showed complete degradation after 7 days. The further investigation of devices using photoluminescence (PL), dark current curves, and electrochemical impedance spectroscopy (EIS), showed this hybrid fullerene surfactant is able to efficiently extract electrons from perovskite absorber to collecting electrodes to result in reduced charge recombination in devices.

**Figure 1:**
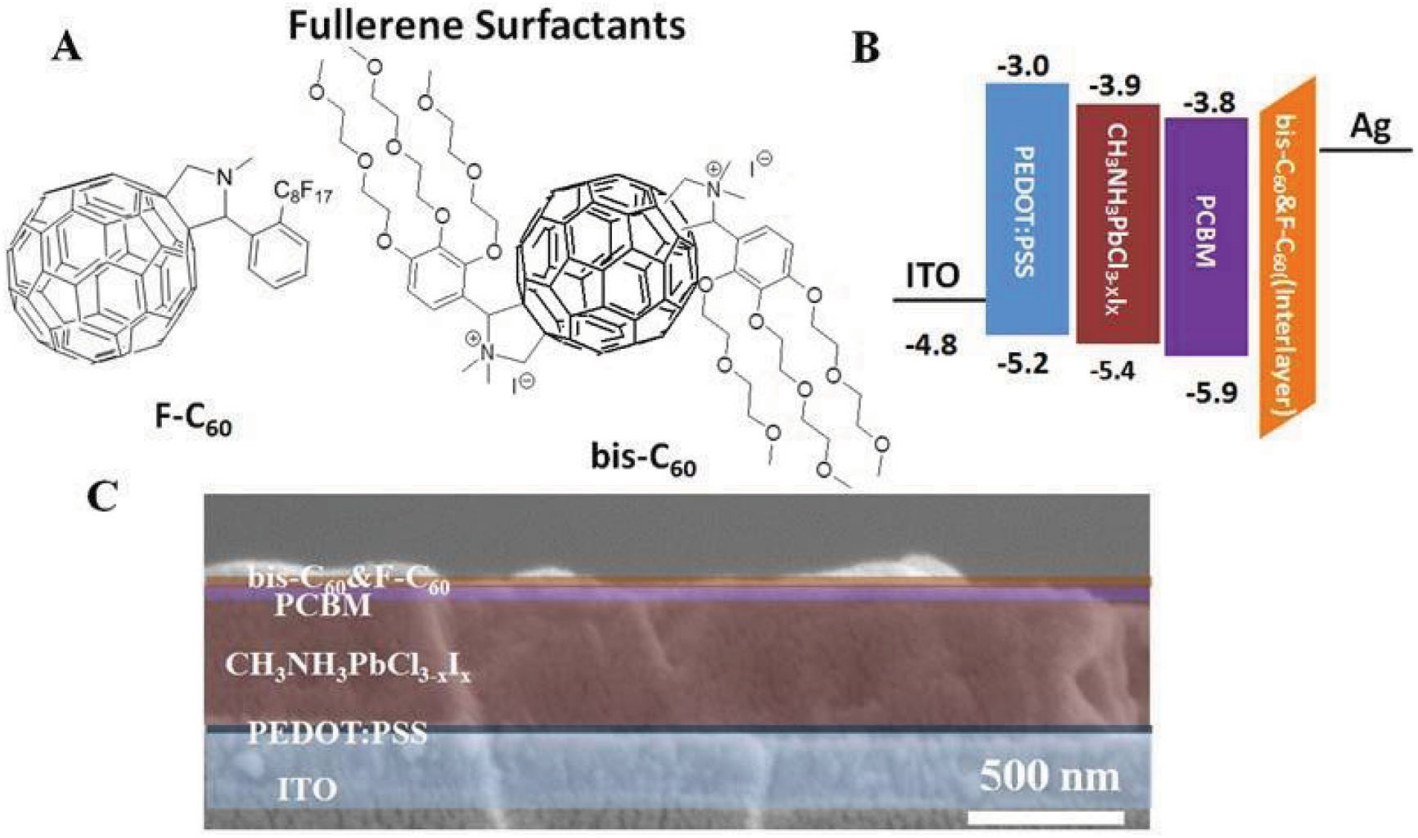
A) Molecular structures of bis‐C_60_ and F‐C_60_. B) Energy level diagram and C) cross‐sectional SEM image of the studied PVSC.

Previously, we have investigated the functions of bis‐C_60_ in organic photovoltaics (OPVs) systematically.[[qv: 15b]] Bis‐C_60_ could be used to tune the work‐function of electrode to enable better aligned energy levels between the organic absorber and electrode to minimize the potential loss across this corresponding interface while also serve as an optical spacer in the stratified device structure to enhance the optical field in organic absorber.^16^ More importantly, bis‐C_60_ is intrinsically conductive because of doping induced by the iodide adjacent to the fullerene core during the film evolution.^17^ Consequently, these merits enable bis‐C_60_ to serve as an efficient FCI in OPVs. Based on this result, we have further explored bis‐C_60_ as an efficient FCI in PVSC to modulate the electrical properties at fullerene/electrode interface to achieve high‐performance thin‐film PVSCs.^18^, ^20^, ^21^

PVSCs are known to be unstable in ambient condition because they are vulnerable to moisture without encapsulation.[[qv: 1a]] It would be very helpful if the hydrophobicity of FCI can be increased to improve the device stability against oxygen and moisture. However, it is critical to maintain good conductivity of the FCI while enhancing its hydrophobicity to improve the performance of derived PVSCs. To achieve this goal, we have synthesized a fullerene derivative (F‐C_60_) with a perfluoroalkyl side‐chain that can be used to blend with bis‐C_60_ to form a hybrid FCI with desirable conductivity (Figure 1A and the detailed synthetic procedure was described in the Experimental Section). The pendent perfluoroalkyl group on F‐C_60_ allows it to have a lower surface energy than bis‐C_60_, which enables it to be diffused onto the top surface of the hybrid FCI during film evolution to minimize moisture penetration into devices.^19^ In order to confirm this, we have measured the contact angel of the studied FCIs as shown in Figure S1 (Supporting Information). It is clearly revealed that F‐C_60_‐based FCIs are more hydrophobic than the pristine bis‐C_60_, suggesting the changed surface composition introduced by the perfluoroalkyl group on F‐C_60_. On the other hand, it is important to note that the similar fullerene moiety in F‐C_60_ and bis‐C_60_ is critical to allow electron delocalization along the packed fullerene networks of both components.

To validate our hypothesis, PVSC with the device configuration of ITO/PEDOT:PSS/CH_3_NH_3_PbCl_3‐_ *_x_*I*_x_*/PCBM/FCI/Ag was fabricated. As shown in Figure 1B, the energy levels of ETL, perovskite layer, and HTL matched well in the corresponding energy level diagram of the derived device. Figure 1C shows the cross‐sectional scanning electron microscope (SEM) image of the PVSC using hybrid FCI to demonstrate the well‐defined mutlilayered structure, where the thickness for PEDOT:PSS, perovskite, PCBM, and FCI is 40, 400, 50, and ≈10 nm, respectively. In addition, a very uniform, thick pervoskite layer can be observed clearly (Figure S2, Supporting Information, without modified color), which is critical for realizing high PCE since it can form a clear p‐i‐n heterojunction to avoid any device shorting.

The current density–voltage (*J–V*) characteristics of studied PVSCs under AM 1.5G irradiation at 100 mW cm^−2^ are shown in **Figure** **2**A and the resulting photovoltaic parameters, such as open‐circuit voltage (*V*_OC_), photocurrent (*J*_SC_), and fill factor (FF), are summarized in **Table** **1**. As shown, the control device without any FCI only had a PCE of 12.1% with a *J*_SC_ of 18.2 mA cm^−2^, a *V*_OC_ of 0.93, and a FF of 71.2%. Consistent with our previous work, employing a bis‐C_60_ FCI can effectively enhance the performance of the derived PVSC to reach a PCE of 14.0% with improved photovoltaic parameters (*J*_SC_: 19.8 mA cm^−2^; *V*_OC_: 0.95; FF: 74.5%), which confirms the advantages of using bis‐C_60_ as discussed previously. However, compared to device with a bis‐C_60_ FCI, the device using only F‐C_60_ FCI merely showed similar performance (PCE: 12.8%; *J*_SC_: 18.7 mA cm^−2^; *V*_OC_: 0.95; FF: 72.2%) as the control device. This is due to the less WF tunability of F‐C_60_ and the lack of self‐doping feature as bis‐C_60_, where the attached PEG side chain and ionic feature of bis‐C_60_ strengthen these unique functions.

**Figure 2:**
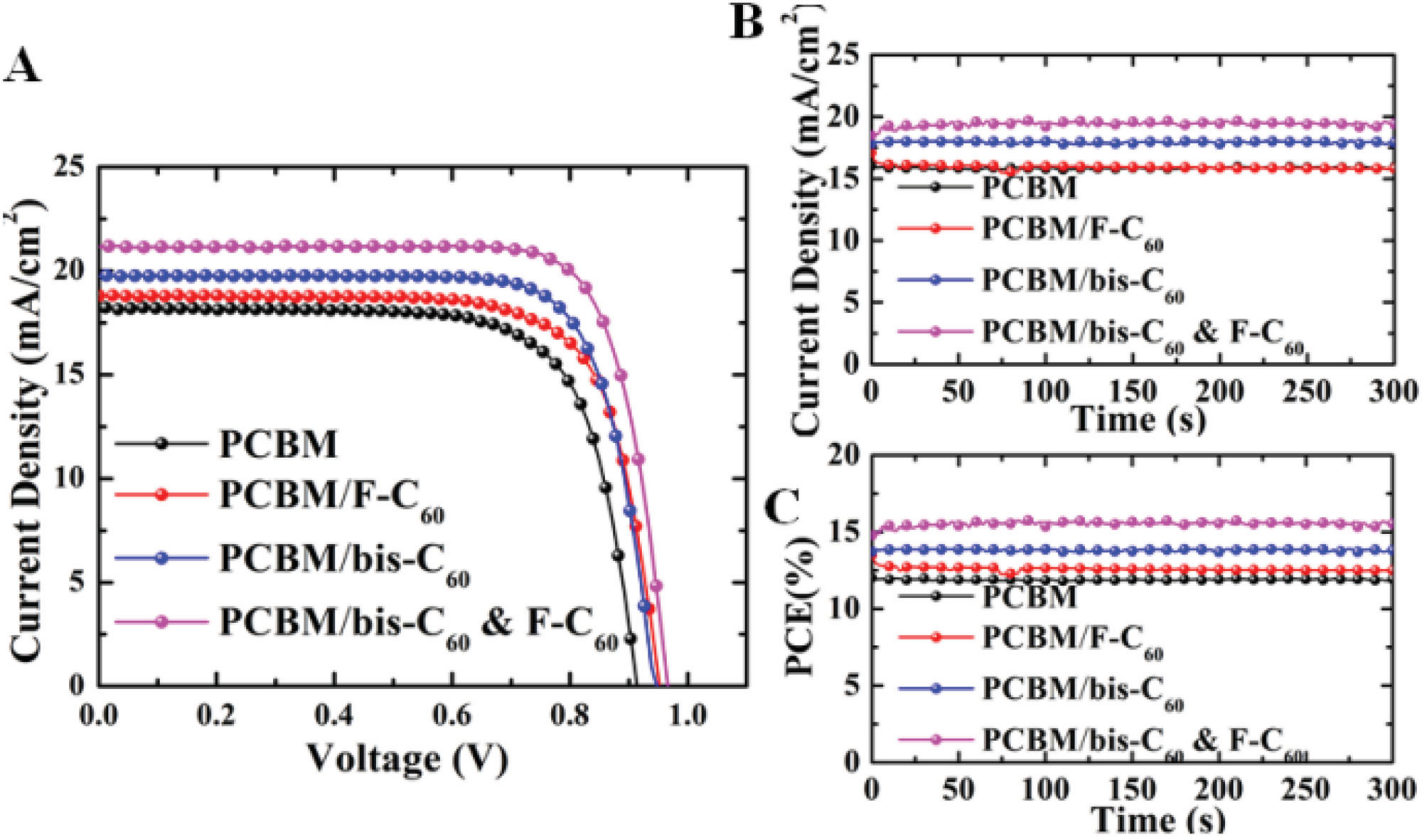
A) Current density–voltage (*J–V*) curves of the PVSCs without and with using the studied FCIs. The stabilized B) photocurrent density and C) PCE measured under a bias near the maximum power point voltage at 0.75 V for without using FCI, 0.75 V for F‐C_60_, 0.77 V for Bis‐C_60_, and 0.80 V for Bis‐C_60_ and F‐C_60_, respectively.

**Table 1:** Photovoltaic performance parameters of solar cells with different scanning direction

ETL	Scan direction	*V*_oc_ [V]	*J*_sc_ [mA cm^−2^]	FF [%]	PCE [%]	PCE average [%]
F‐C_60_+bis‐C_60_	Forward	0.97	21.2	75.4	15.5	15.3
	Reverse	0.97	20.7	75.2	15.1	
bis‐C_60_	Forward	0.95	19.8	74.5	14.0	13.8
	Reverse	0.95	19.4	74.1	13.6	
F‐C_60_	Forward	0.95	18.7	72.2	12.8	12.7
	Reverse	0.95	18.6	71.8	12.6	
None (PCBM)	Forward	0.93	18.2	71.2	12.1	11.9
	Reverse	0.92	18.1	70.5	11.7	

By blending F‐C_60_ with bis‐C_60_, it enhances the hydrophobicity of the FCI, which in turn increases ambient stability of the derived PVSCs and maintains the appealing electrical conductivity of bis‐C_60_. As shown in Figure 2A, the derived PVSC yielded the highest PCE of 15.5%, with a *V*_OC_ of 0.97 V, a *J*_SC_ of 21.2 mA cm^−2^, and a FF of 75.4%. To further examine the reliability of these studied PVSCs, the stabilized photocurrents measured under a bias near the maximum power point of the corresponding devices were conducted and shown in Figure 2B. The stabilized power outputs were calculated by multiplying the photocurrent and photovoltage. As shown in Figure 2C, a steady‐state PCE of 15.4% which is very close to the PCE measured, can be obtained for the top‐performing PVSC using a hybrid FCI, demonstrating the high reliablity of these PVSCs. In addition, the calculated average PCEs of the devices were in good agreement with the stabilized power outputs. The external quantum efficiency (EQE) spectra of the devices are shown in Figure S3 (Supporting Information), where the integrated current correlates well with the corresponding *J*_SC_ values shown in Figure 2A. Besides, Figure S4 (Supporting Information) and Table 1 depicted the four *J–V* curves and detailed parameters for the PVSCs with or without using the studied FCIs. Negligible hysteresis is observed, which can be attributed in part to the effective electronic coupling at perovskite/PCBM interface.

Ambient stability of the PVSCs is a major concern for the widespread deployment of this technology.^20^ Previous studies showed that the photodegradation of a device with a configuration of PCBM/bathocuproine/Al is even faster than that of the perovskite layer itself.^13^ This might be due to the incomplete coverage of PCBM on perovskite film to result in water/oxygen absorption as mentioned earlier.[[qv: 12b]] To compare the suitability of employing this new hybrid FCI to improve ambient stability, we have conducted a parallel study with other devices without FCI or only using a single FCI. All devices were exposed in ambient condition with 20% relative humidity without encapsulation. All of the exposed PVSCs were then tested every 24 h under one‐sun illumination in the nitrogen glovebox.

**Figure** **3** illustrates the device parameters of PCE, *J*_SC_, *V*_OC_, and FF as a function of storage time in ambient condition. As seen in Figure 3A, the control device degraded quickly to zero after 168 h while the device using a bis‐C_60_ FCI decayed to zero after 336 h. It can be inferred that the bis‐C_60_ FCI is beneficial to improve the coverage of fullerene onto perovskite layer to retard the penetration of oxygen or moisture into perovskite layer through ETL. Very encouragingly, the ambient stability of the device using the hybrid FCI can be dramatically improved and preserve ≈80% of its original efficiency after 336 h (two weeks) of exposure in ambient condition. The standard measurement condition of lifetime test at a relative humidity of 85% is also investigated as shown in Figure S5 (Supporting Information). Despite the accelerated degradation of all studied PVSCs with the increased ambient humidity, the devices using the top cathode interlayers containing F‐C_60_ clearly deliver better stability than the device only using bis‐C_60_. This result affirms the advantage of introducing hydrophobic perfluoroalkyl groups to effectively prevent water penetration into the perovskite films as a result of the enhanced hydrophobicity introduced by F‐C_60_ (Figure S1, Supporting Information). All these results demonstrate that by simply introducing long perfluoroalkyl side‐chains on the fullerene core, it increases the hydrophobicity and thereby effectively blocks the moisture from penetrating into the devices. This result shows the feasibility of enhancing air stability of PVSCs via rationally molecular design of employed cathode interlayer.

**Figure 3:**
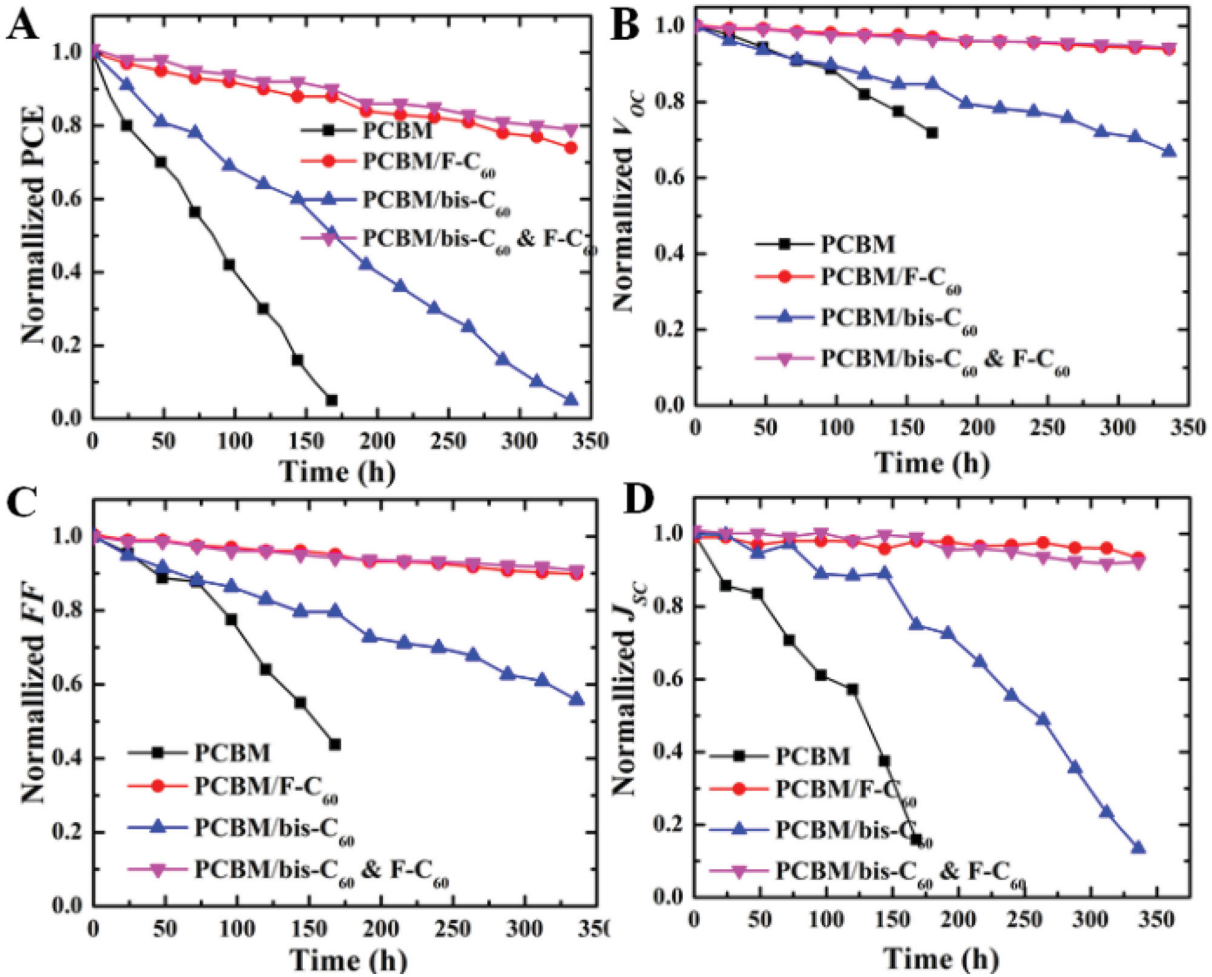
Normalized A) PCE, B) *V*_OC_, C) *J*_SC_, and D) FF of PVSCs without and with using the studied FCIs as a function of storage time in ambient condition (air) with 20% humidity.

As revealed eariler, the device using the hybrid FCI even showed an improved PCE compared to the device using only pristine bis‐C_60_. To elucidate the origin of this enhancement, we have further examined the charge‐transporting properties in these devices by using PL and impedance spectroscopies. We first measured and compared the steady state PL spectra of pristine perovskite, perovskite/PCBM, and perovskite/PCBM/FCIs on the ITO substrates. The thicknesss of all layers (perovskite, PCBM, and surfactants) were kept the same as the optimized thicknesses used in the devices. All these samples exhibit a PL peak at 760 nm, which results from CH_3_NH_3_PbCl_3‐_ *_x_*I*_x_*.^21^] **Figure** **4**A showed clearly the quantum yield of perovskite PL was significantly reduced after sequential deposition of PCBM and surfactants. This PL quenching is attributed to the charge carrier extraction from CH_3_NH_3_PbCl_3‐_ *_x_*I*_x_* to fullerenes. Notably, the degree of PL quenching is strongly correlated with employed surfactants. It is found the PL quenching efficiency followed the orders of PCBM < PCBM/F‐C_60_ < PCBM/bis‐C_60_ < PCBM/hybrids. This result indicates that the FCIs might reduce the space charges to further enhance the charge dissociation due to their proper electron mobility.^22^ We have also measured the electron mobility of the studied FCIs by fabricating the electron‐only devices with the configuration of ITO/ZnO/FCIs/LiF/Al. As shown in Figure S6 and Table S1 (Supporting Information), the bis‐C_60_ possesses the highest electron mobility of 20.6 × 10^−4^ cm^2^ V^−1^ s^−1^, the hybrid FCI has a slightly lower value of 10.7 × 10^−4^ cm^2^ V^−1^ s^−1^, while the pristine F‐C_60_ shows the lowest electron mobility of (3.2 × 10^−4^ cm^2^ V^−1^ s^−1^). This result affirms that the potential of hybrid FCI to yield comparable or even better photovoltaic performance than pristine bis‐C_60_.

**Figure 4:**
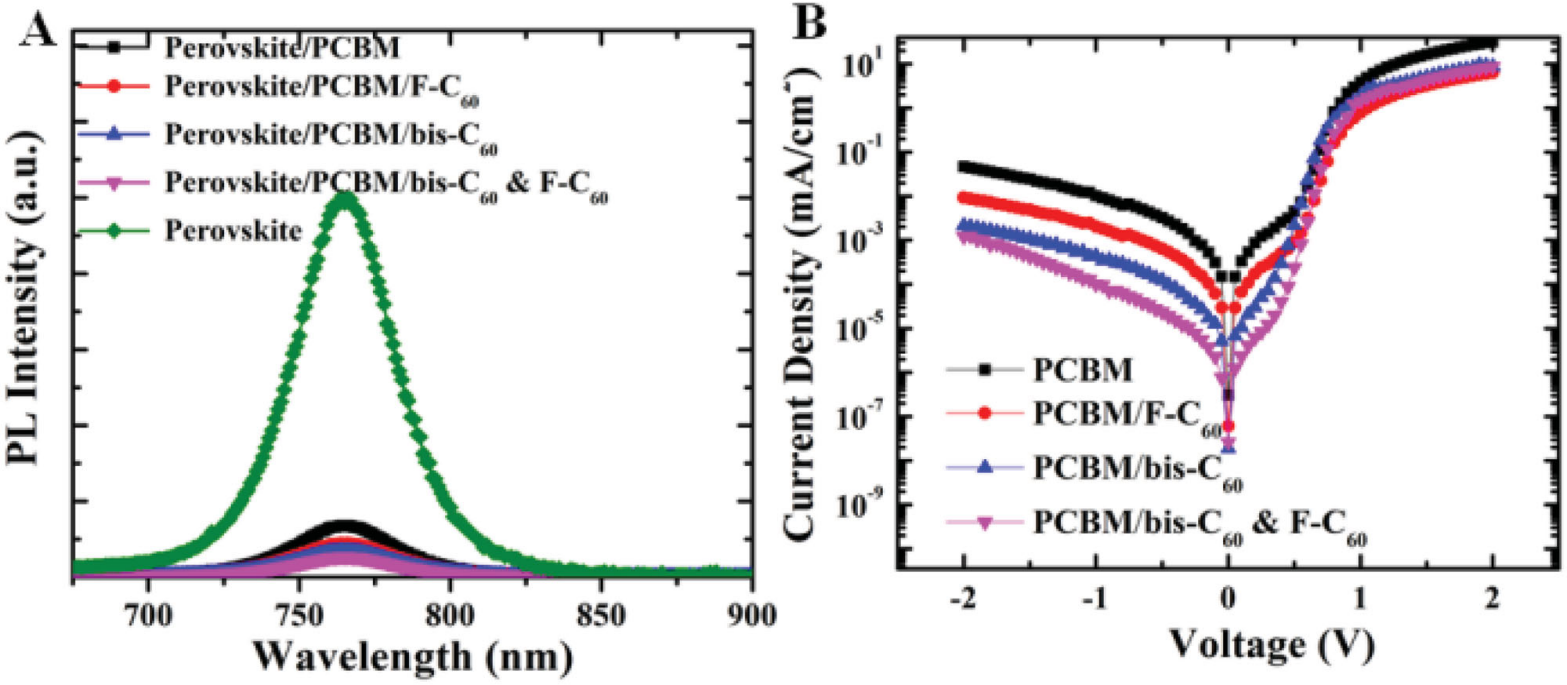
A) Steady‐state PL spectra of perovskite (green line), perovskite/PCBM (black line), perovskite/PCBM/surfactants (F‐C_60_ (red line), Bis‐C_60_(blue line), and hybrid (magenta line)). B) Dark currents of the studied PVSCs.

Since dark current measurements is an effective method to evaluate charge carrier loss through the leakage pathways and the recombination of free carriers in the device during operating condition,^23^ we have also measured the dark current of the studied PVSCs and the related *J–V* curves as shown in Figure 4B. As can be seen, the dark current density of the devices using the hybrid FCI is two orders lower than that of the control device. Moreover, the device using the respective bis‐C_60_ and F‐C_60_ FCI also showed a much lower PL intensity compared to the control device. These results illustrated that the FCIs could effectively decrease the current leakage throughout the cell and reduce the recombination of charge carriers between the electrode and PCBM ETL. This confirms that the electronic properties at this corresponding interface are mediated by the fullerene surfactant as discussed previously.

EIS has been widely used to measure the charge transport process in solar cells, such as chemical capacitance, recombination resistance, charge conductivity, etc.^24^, ^25^ To further understand the charge transfer behavior in our devices, especially the charge recombination from the PCBM ETL to the perovskite layer, we have measured the devices under operating conditions by the EIS.^22^] **Figure** **5** showed the Nyquist plots of the devices without or with using the studied FCIs, which were recorded at different applied voltages under illumination. The frequency used for all of the EIS measurements ranges from 0.5 Hz to 1 MHz. All devices were applied with a bias ranging from 0 to 0.9 V. The obtained data were fitted based on a comonly used equivaltent circuit model as shown in Figure 5A. According to the previous EIS studys for PVSCs, the typical Nyquist plots can be demarked into two main regions/arcs at high and low frequencies. The arc at high frequencies can be assigned to the carrier diffusion through the device while the low frequencies's arc represents the recombination process in device. Therefore, for the sake of convenience, a parallel transport resistance (*R*_trans_) – chemical capacitance (*C*_trans_) subcircuit was applied, while *R*_rec_ and *C*_rec_ represent the recombination resistance and capacitance, respectively. Figure 5F showed the comparison of *R*_rec_ of the PVSCs using different FCIs. The results indicated that the order of *R*_rec_ is *R*_rec_(PCBM) < *R*_rec_(PCBM/bis‐C_60_) < *R*_rec_(PCBM/F‐C_60_) < *R*_rec_(PCBM/hybrid). Accordingly, the recombination lifetime (*τ*_rec_) can be derived from the equation of *τ*_rec_ = *R*_rec_ *C*_rec_. The estimated *τ*_rec_ is in the similar order of *τ*_rec_(PCBM) < *τ*_rec_(PCBM/bis‐C_60_) < *τ*_rec_(PCBM/F‐C_60_) < *τ*_rec_(PCBM/hybrid). This result proves that the recombination pathway from metal electrode to perovskite layer can be effectively suppressed by these additional FCIs. This confirms the function of the hybrid FIC in enhancing charge collecting efficiency and reducing charge recombination for improved device performance.

**Figure 5:**
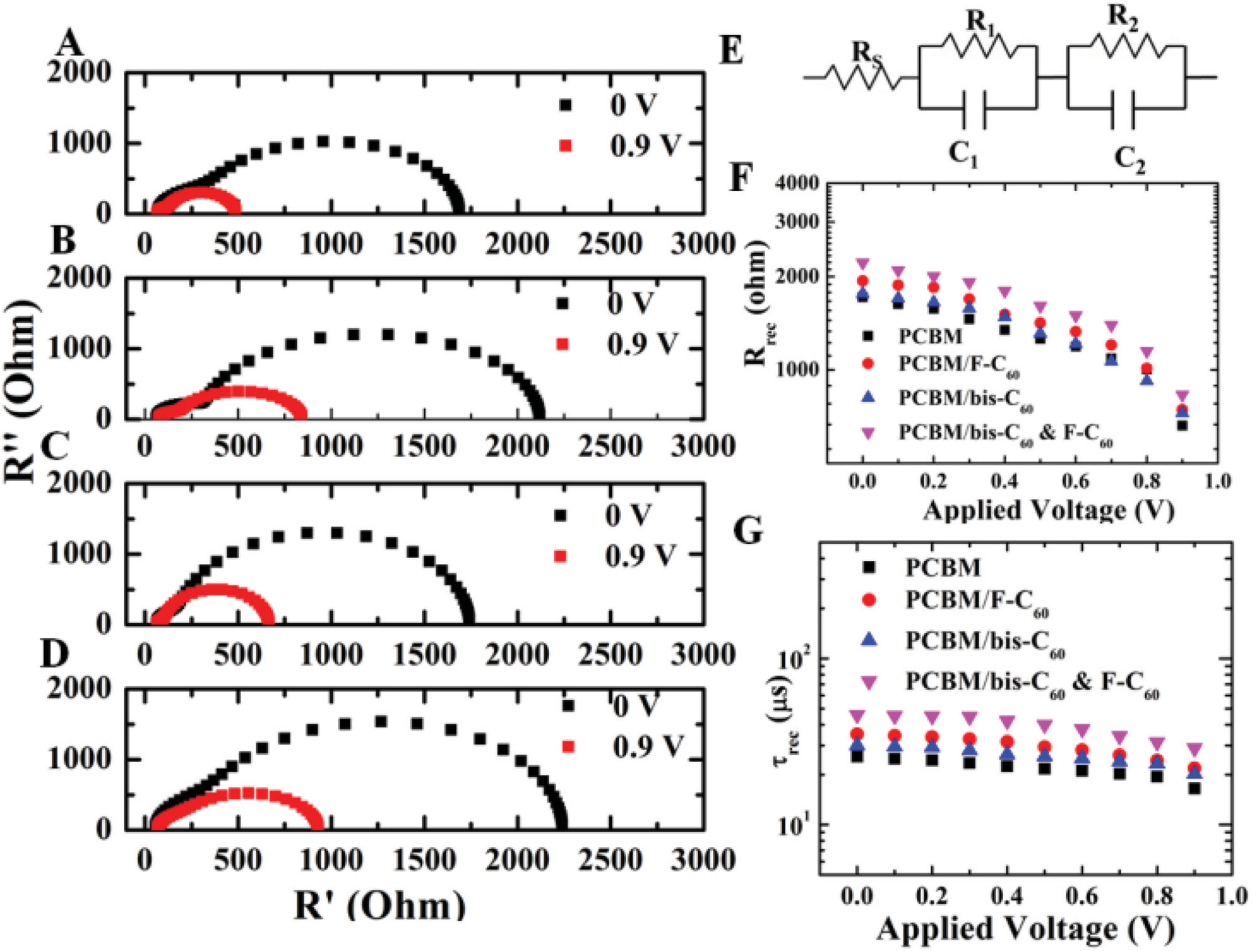
Impedance spectroscopy characterization. The representative Nyquist plots of whole regions of impedance spectra at different biases (short‐circuit 0 V and applied bias voltage 0.9 V) A) without interlayer (None) and with interlayer (B)F‐C_60_, C) Bis‐C_60_, and D) Bis‐C_60_, and F‐C_60_) under illumination. The equivalent circuit E) used for fitting the Nyquist plots. Plots a series of characteristic recombination resistance F) and recombination lifetime G) from impedance spectroscopy measurements obtained from the cells at different bias voltage under illumination.

In summary, we have demonstrated a high‐performance PVSC with good ambient stability by employing a hybrid FCI decorated with perfluoroalkyl side‐chains. This hybrid FCI was found to enhance charge collection from perovskite to electrode and impede the charge recombination in the active layer as evidenced from the PL, dark current test, and EIS spectroscopies. As a result, an improved PCE (from 12.1% to 15.5 %) and ambient stability can be achieved. The device can retain 80% of its original PCE even after being exposed in ambient condition with 20% relative humidity for two weeks without any encapsulation. This work demonstrates a simple and generally applicable strategy to enhance the ambient stability of PVSCs *via* rationally molecular design of cathode interlayer.

## Experimental Section

*General Methods*: All chemicals and reagents were used as received from commercial sources without purification. Solvents for chemical synthesis were purified by distillation. All chemical reactions were carried out under an argon or nitrogen atmosphere. Bis‐C_60_ was synthesized according to previous procedure.[[qv: 15b,c]]] ^1^H, ^13^C, and ^19^F NMR spectra were recorded on a Bruker AV500 spectrometer.

*Synthesis of N‐Methyl‐2‐(2‐perflurooctylphenyl)‐3,4‐fulleropyrrolidine (F‐C_60_)*: A solution of C_60_ (288 mg, 0.40 mmol), 2‐perfluorooctylbenzaldehyde (273 mg, 0.52 mmol), and *N*‐methyl glycine (61 mg, 0.68 mmol) in 40 mL anhydrous chlorobenzene was heated to reflux under argon for 8 h. The reaction mixture was then cooled to room temperature. After removal of the solvent, the crude product was purified by column chromatography on silica gel with toluene as eluent to afford F‐C_60_ as a brown powder (239 mg, 47%). ^1^H NMR (CDCl_3_, 500 MHz) δ 8.66 (d, *J* = 10 Hz, 1H), 7.71 (t, *J* = 5 Hz, 1H), 7.65 (d, *J* = 10 Hz, 1H), 7.50 (t, *J* = 5 Hz, 1H), 5.38 (s, 1H), 4.98 (d, *J* = 10 Hz, 1H), 4.32 (d, *J* = 10 Hz, 1H), 2.72 (s, 3H); ^13^C NMR (CDCl_3_, 125 MHz) δ 156.33, 153.73, 153.66, 153.23, 147.37, 146.43, 146.27, 146.16, 146.08, 146.00, 145.65, 145.59, 145.54, 145.46, 145.39, 145.26, 145.22, 145.15, 144.65. 144.48, 144.42, 143.14, 143.06, 142.65, 142.57, 142.32, 142.21, 142.09, 142.02, 141.74, 141.67, 141.55, 140.25, 140.18, 139.79, 139.45, 136.38, 136.09, 132.75, 132.33, 128.74, 128.56, 78.19, 69.45, 39.23; ^19^F NMR (CDCl_3_, 470 MHz) δ −80.68, −100.04, −119.83, −120.92, −121.63, −122.57, −126.00; HRMS (m/z, MALDI) Calcd for C_77_H_10_F_17_N 1271.0542, found 1271.0497. ^1^H, ^13^C, and ^19^F NMR spectra were shown in Figures S7–S9 (Supporting Information).

*Solar Cell Fabrication and Characterization*: PCBM solution was prepared by dissolving PCBM into chlorobenzene with a concentration of 20 mg mL^−1^. Bis‐C_60_ and F‐C_60_ surfactant were prepared 2 mg mL^–1^ in isopropyl alcohol respectively. The recipes of the prepared bis‐C_60_ and hybrid surfactant (weight ratio of bis‐C_60_ and F‐C_60_: 7:3) solutions are all 2 mg mL^−1^ in isopropyl alcohol while that for F‐C_60_ solution is 1 mg mL^−1^. All the solutions were ultra‐sonicated for 3 h and then filtered with a 0.2 μm filter prior to spin‐coating. The exact concentration of the filter surfactant solution is about 1.8 mg mL^−1^ for bis‐C_60_, 0.5 mg mL^−1^ for F‐C_60_. For the hybrid surfactant solution, the concentration of bis‐C_60_ and F‐C_60_ is 1.2 mg mL^−1^ and 0.4 mg mL^−1^, respectively. CH_3_NH_3_I was synthesized under an ice bath for 2 h by reacting methylamine (CH_3_NH_2_, 33 wt% in ethanol from Sigma‐Aldrich) with hydroiodic acid (HI, 57 wt% in water from Sigma‐Aldrich). The white powders were precipitated by drying at 60 °C and washed for three times with diethyl ether (Sigma‐Aldrich) before further dried out to be stored in nitrogen‐filled glove‐box. CH_3_NH_3_PbI_3‐_ *_x_*Cl*_x_* precursor solution was prepared by dissolving Lead iodide (99.999%, Alfa Aesar), lead chloride (99.999%, Alfa Aesar), and CH_3_NH_3_I in anhydrous *N,N*‐Dimethylformamide (DMF, 99.8%, Sigma‐Aldrich) as molar ratio 1:1:4 with concentration of 40 wt%.

The ITO‐coated (15 Ω sq^–1^) glass substrates were cleaned and treated with UV ozone for 20 min. Then PEDOT:PSS (Baytron 4083) was spin‐coated onto ITO substrates at 4000 r.p.m. for 30 s. Then after heating at 100 °C for 10 min, the perovskite precursor solution was spin‐coated at 3000 r.p.m. for 30 s. After drying for more than 10 min, the as spun films were annealed over 50 min at 100 °C in the case of CH_3_NH_3_PbI_3‐_ *_x_*Cl*_x_*.Then, the PCBM electron transport layer was deposited by spin coating at 1500 r.p.m for 60 s and 2000 r.p.m for 5 s. Afterward, surfactant layer was spin‐coated at 3000 r.p.m for 30 s. Finally, a 150‐nm thick top Ag electrode was evaporated under high vacuum (<2 × 10^−6^ Torr). For all devices, the active area was defined as 3.14 mm^2^ by shadow mask. All the *J–V* curves in this study were recorded by using Keithley 2400 source measurement unit and the scan rate was kept at 0.1 V s^–1^. The photocurrent was measured under illumination from a 450 W thermal Oriel solar simulator (AM 1.5G). The illumination intensity of the light source was accurately calibrated employing a standard Si photodiode detector equipped with a KG‐5 filter, which can be traced back to the standard cell of National Renewable Energy Laboratory. We measured the EIS spectra at an applied bias of *V*_oc_ and a frequency range from 0.5Hz and 1MHz with AC amplitude of 10 mA under illumination. Z‐View Analyst software was used to model the Nyquist plots obtained from the impedance measurements. The SCLC mobility (μ) is evaluated by fitting the dark *J–V* characteristics with the Mott–Gurney equation for the current density *J*_SCLC_ expressed as

(1) $$J=\frac{9\varepsilon_{r}\varepsilon_{0}\mu V^{2}}{8L^{3}}$$

where *ε*_0_ is the vacuum permittivity, *ε*_r_ is the dielectric constant of the film (*ε*_r_ = 3 was assumed), and *L* is the thickness of the active layer.

## Supporting information

As a service to our authors and readers, this journal provides supporting information supplied by the authors. Such materials are peer reviewed and may be re‐organized for online delivery, but are not copy‐edited or typeset. Technical support issues arising from supporting information (other than missing files) should be addressed to the authors.

Supplementary
