# Supplementary material for: Enhanced Ambient Stability of Efficient Perovskite Solar Cells by Employing a Modified Fullerene Cathode Interlayer
Source: Adv Sci (Weinh). 2016 Mar 22;3(9):1600027. doi: 10.1002/advs.201600027 (PMC5039977; doi:10.1002/advs.201600027)
Supplement: Supplementary file 1 — Supplementary [file ADVS-3-0a-s001.pdf]

Copyright WILEY-VCH Verlag GmbH & Co. KGaA, 69469 Weinheim, Germany, 2016.

ADVANCED SCIENCE
Open Access

Supporting Information

for *Adv. Sci.*, DOI: 10.1002/advs.201600027

# Enhanced Ambient Stability of Efficient Perovskite Solar Cells by Employing a Modified Fullerene Cathode Interlayer

*Zonglong Zhu, Chu-Chen Chueh, Francis Lin, and Alex K.-Y. Jen**

Supporting Information

**Enhanced Ambient Stability of Efficient Perovskite Solar Cells by Employing a Novel Fullerene Cathode Interlayer**

*Zonglong Zhu[1], Chu-Chen Chueh[1], Francis Lin[2], Alex K.-Y. Jen[1,2*]*

[1]Department of Materials Science and Engineering, [2]Department of Chemistry, University of Washington, Seattle, WA 98195, USA

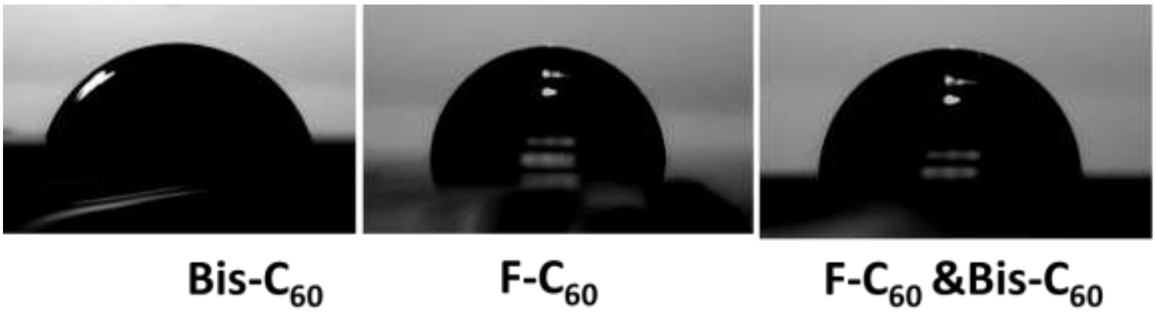


**Figure S1.** Contact angle measurement of bis-$C_{60}$, F-$C_{60}$ and hybrid surfactant (bis-$C_{60}$ & F-$C_{60}$), wherein the contact angle is $68^o$ for bis-$C_{60}$, $96^o$ for F-$C_{60}$, and $84^o$ for bis-$C_{60}$ & F-$C_{60}$.

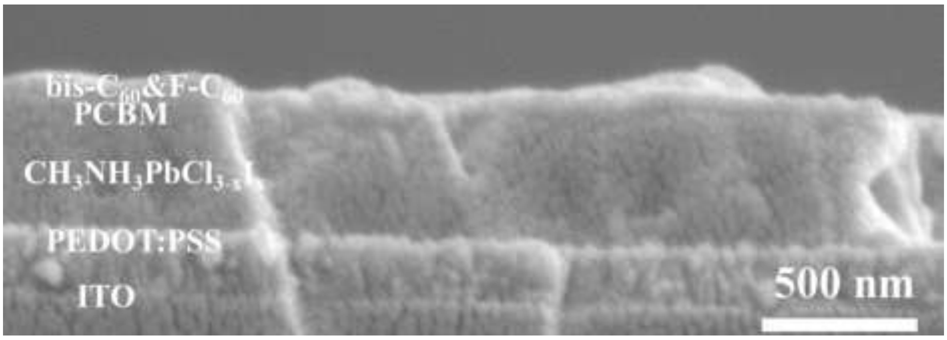


**Figure S2.** Cross-section SEM image of the top-performing PVSC in this study.

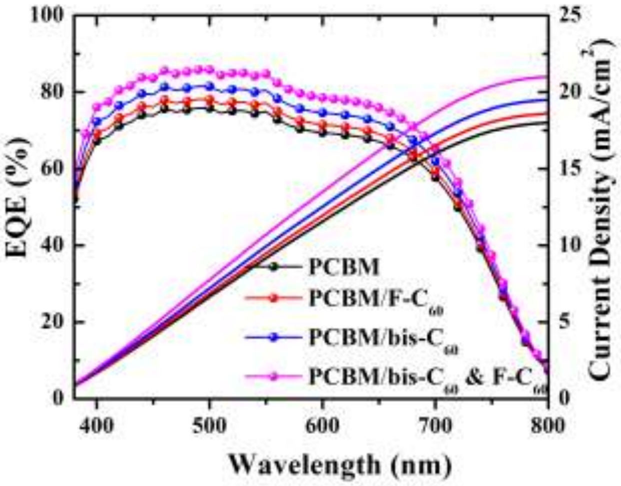


**Figure S3.** The EQE spectra and their integrated photocurrent densities of the IPCE spectra of perovskite solar cells without interlayer (None) and with interlayer ((B)F-$C_{60}$, (C) Bis-$C_{60}$, and (D) Bis-$C_{60}$&F-$C_{60}$)

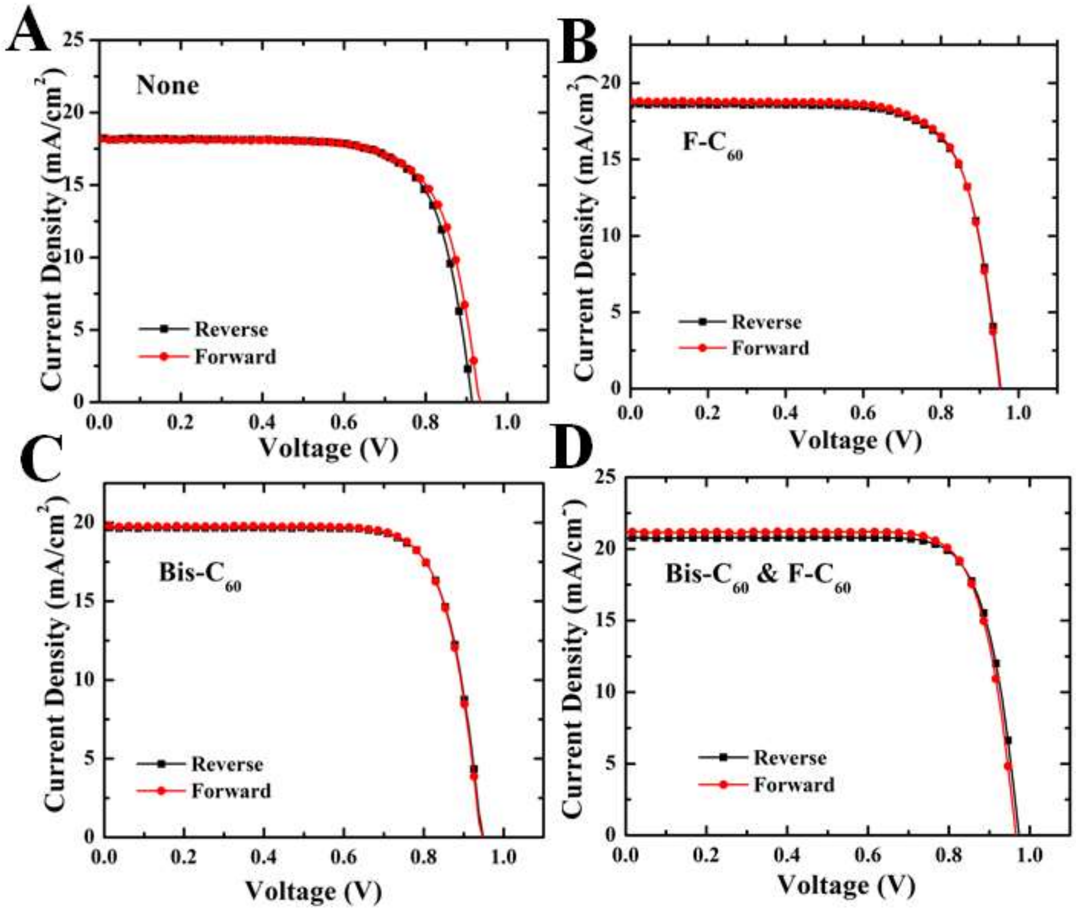


**Figure S4**. J-V curves for (A) without interlayer (None) and with interlayer ((B)F-$C_{60}$, (C) Bis-$C_{60}$, and (D) Bis-$C_{60}$&F-$C_{60}$) which measured by forward (from short circuit to open circuit) and reverse (from open circuit to short circuit) scans. All J-V curves were measured under 100 mW/$cm^2$ air mass 1.5 global (AM 1.5G) illumination.

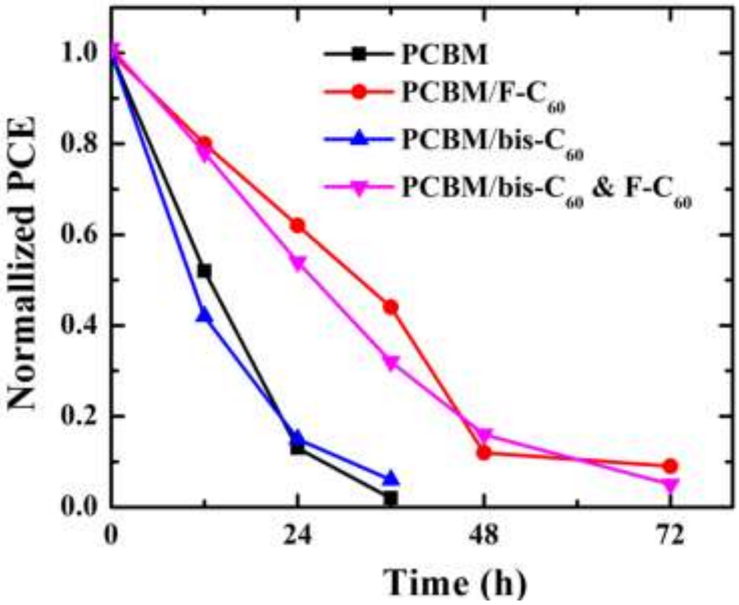


**Figure S5.** Normalized PCE, of PVSCs without and with using the studied FCIs as a function of storage time in ambient condition (air) with a relative humidity of 85 %.

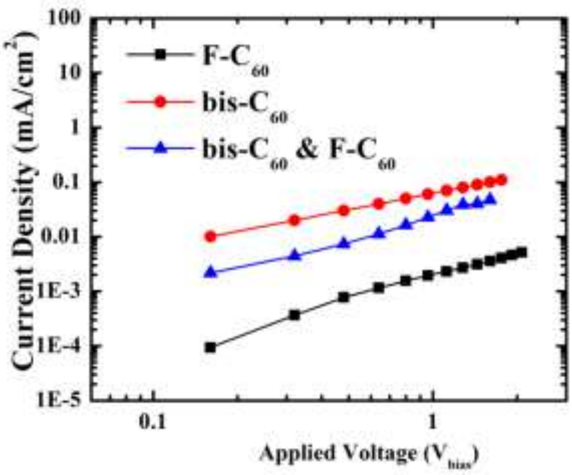


**Figure S6**. Measured space-charge-limited *J-V* characteristics of the electron-only devices (ITO/ZnO/fullerene surfactants/LiF/Al) under dark conditions.

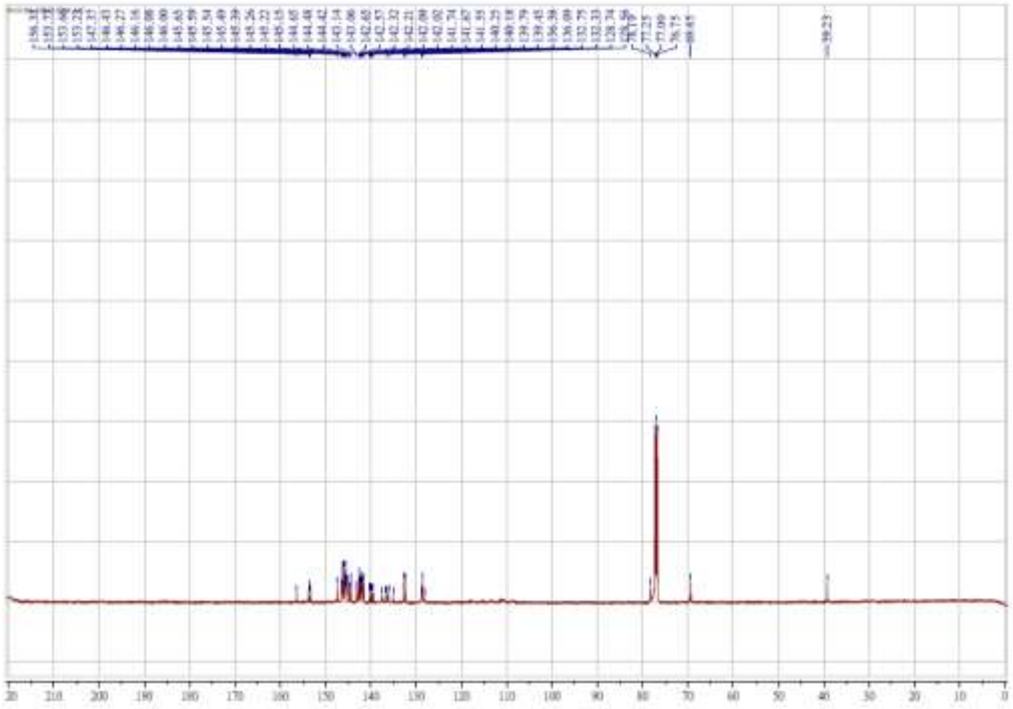

**Figure S7**. $^{13}$C NMR spectra of F-$C_{60}$.

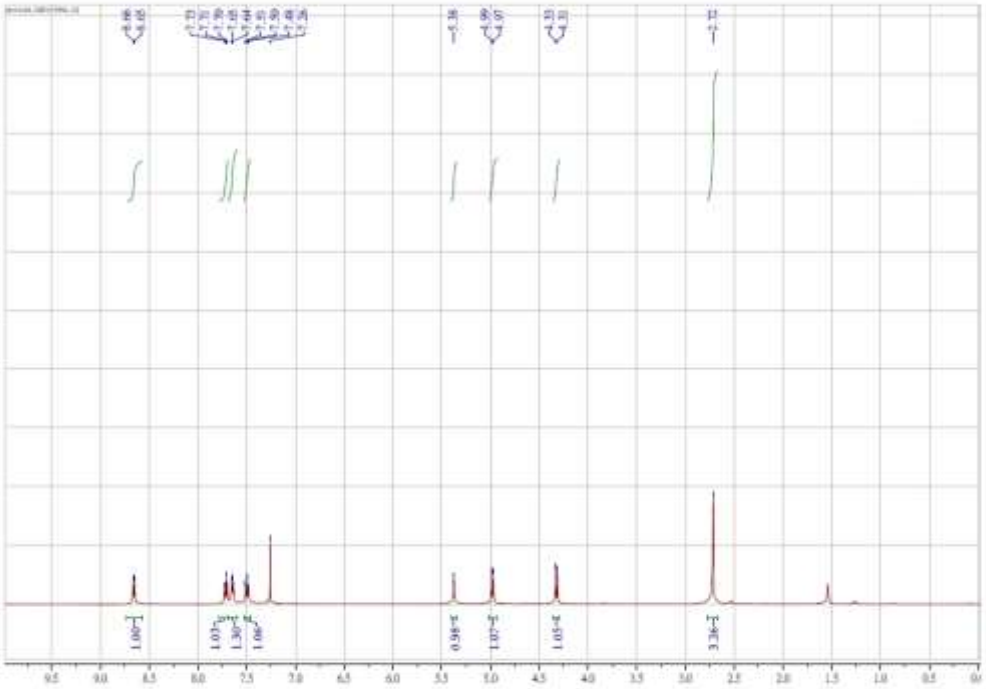

**Figure S8.** $^{1}$H NMR spectra of F-$C_{60}$

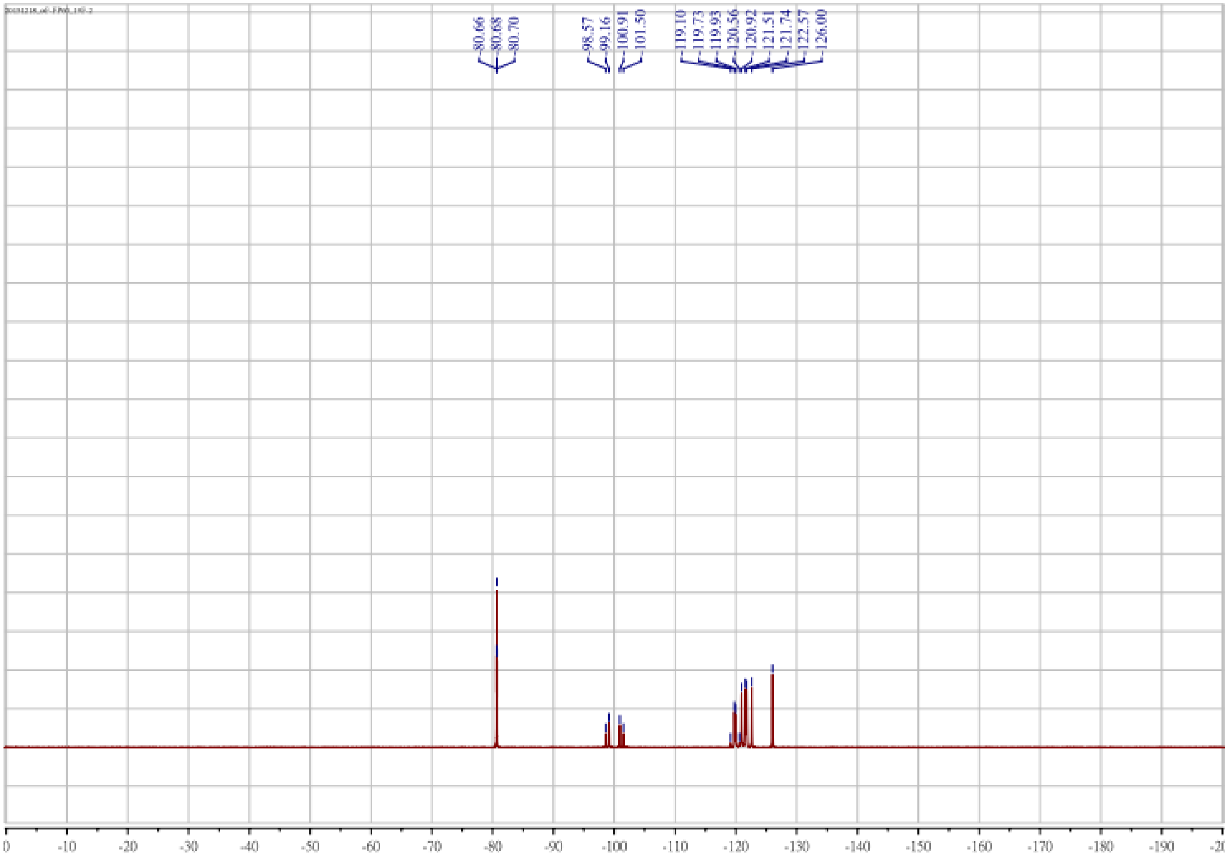

**Figure S9**. $^{19}$F NMR spectrum of F-$C_{60}$.

**Table S1**. The estimated SCLC electron mobility of the fullerene surfactants.

| | **Bis-$C_{60}$,** | **F-$C_{60}$** | **Bis-$C_{60}$ & F-$C_{60}$** |
|---|---|---|---|
| Mobility ($\times 10^{-4}$ $cm^2$ $V^{-1}s^{-1}$) | 20.6 | 3.2 | 10.7 |
| Thickness (nm) | 100 | 100 | 110 |
